# Distribution of Metals During Carbothermic Reduction of Antimony from Sodium Antimonate

**DOI:** 10.3390/ma19091848

**Published:** 2026-04-30

**Authors:** Valeriy Volodin, Bagdaulet Kenzhaliyev, Sergey Trebukhov, Alina Nitsenko, Farkhad Tuleutay, Xeniya Linnik, Bulat Sukurov

**Affiliations:** Institute of Metallurgy and Ore Beneficiation, Satbayev University, 050010 Almaty, Kazakhstan; volodinv_n@mail.ru (V.V.); bagdaulet_k@satbayev.university (B.K.); s.trebukhov@satbayev.university (S.T.); farkhat_kaldybek@mail.ru (F.T.); xenija_linnik@mail.ru (X.L.); bsukurov@gmail.com (B.S.)

**Keywords:** sodium antimonate, antimony, distribution, carbothermic reduction, coke, sodium, lead, crude metal, slag

## Abstract

In this study, the carbothermic reduction of sodium antimonate in crucible smelting was investigated. The optimal process temperature was determined to be 900 °C, with 10% coke consumption (with an ash content up to 15.33%) and a feed particle size of minus 1 mm. The process does not involve the addition of slag-forming components. Sodium participates in the formation of the slag phase. According to the smelting results, the amount of antimony recovered as crude metal reached 71–72%, while the Sb content in the crude metal reached up to 94.5%. A significant portion of antimony (up to 27%) volatilizes with off-gases. A notable sodium content was detected in the crude antimony, reaching up to 8% in some samples, while more than 80% of sodium was transferred to the slag phase. Arsenic, present in the initial concentrate at a level of 0.6%, was distributed approximately equally among the metallic, slag, and gas phases. Lead was predominantly concentrated in the crude antimony. Iron preferentially dissolved in the crude antimony. Other impurities were distributed in comparable amounts between the metallic and slag phases. Tellurium, present in sodium antimonate at 0.79%, was detected in some samples within the slag phase.

## 1. Introduction

Currently, the countries with the largest antimony reserves are China, Russia, Bolivia, and Kyrgyzstan, each accounting for more than 10% of global reserves [1]. The remaining countries, accounting for less than 10% each, collectively account for approximately 30% of global reserves. The Republic of Kazakhstan does not possess significant antimony deposits.

Antimony is widely used across various industries because of its ability to enhance the hardness of alloyed materials, the flame-retardant properties of its compounds, and its semiconductor characteristics. Therefore, antimony is an important industrial element primarily used in metallurgy, the production of flame-retardant materials, batteries, electronic devices, and the chemical industry [2,3,4]. The United States and the European Union have classified antimony as a critical raw material [5,6], and, by 2050, it is expected to become one of the scarcest metals. In this regard, researchers and engineers are increasingly directing their attention toward the recovery of antimony from waste materials and the intermediate products of metallurgical processes [7,8,9,10,11,12,13].

One such source of antimony is sodium antimonate, an intermediate product in the technological process of crude-lead refining [14]. Due to the lack of metallic antimony production in Kazakhstan, this intermediate product has not been smelted separately in domestic plants, and this remains the case today. However, technological studies on the processing of sodium antimonate with the recovery of rare metals were conducted in Kazakhstan in the late 1950s, primarily at the Shymkent Lead Plant [15,16,17,18,19]. In recent years, research interest in the processing of sodium antimonate has been renewed, particularly due to its significant generation within the technological scheme of large-scale lead production. Both hydrometallurgical [20,21] and pyrometallurgical processing methods [22,23,24,25] are currently being developed, with preference generally given to high-temperature reduction processes.

Reduction smelting leverages the relatively easy reducibility of antimony oxides at temperatures of 800–1000 °C [24], using solid carbon (up to 40% of the antimony-containing feed mass) and carbon monoxide as reducing agents. Soda is commonly used as a flux, enabling the formation of a low-melting-point slag (comprising approximately 45% Na_2_O and less than 20% SiO_2_). As a result of smelting, crude antimony containing 96–99% Sb can be obtained, with recovery levels of approximately 90%.

Reduction smelting is carried out in reverberatory, short-rotary-drum, rotary, and shaft furnaces. Studies have also been conducted on the application of electric smelting for oxidized antimony materials [26,27,28]. The antimony content in such materials, primarily present as antimony trioxide, ranges from 28 to 36%. In these studies, smelting was performed with the addition of 35–52% soda (relative to the antimony-containing material) and 5–10% coke. The process was conducted at temperatures of 1200–1300 °C. Under these conditions, direct recovery of antimony as crude metal reached 70–82%, with 3–5% reported to be slag and 14–24% volatilizing with off-gases. When recycled dust is processed, total antimony recovery as crude metal can reach up to 95.5% [28].

Reduction smelting of sodium antimonate to produce commercial-grade antimony has also been investigated [29,30]. The objective of these studies was to determine optimal process parameters for processing antimony concentrate (sodium antimonate) to obtain marketable antimony within the existing technological infrastructure of lead pyrometallurgical production [29]. The optimal charge composition was determined as follows: 100% sodium antimonate and 9% coke. This composition ensured the maximum yield of the metallic phase (40–45%) and a minimum slag yield (29–45%). The use of fluxes for smelting antimony concentrate was not recommended, as the process is sustained by the presence of sodium salts in the antimonate and soda formed in the slag. According to the authors, processing 2000 tons of sodium antimonate with subsequent antimony refining can generate an annual profit of approximately 6 million rubles (about US$78,000).

The authors of [31] studied the reduction of antimony concentrate in alkaline melts under carbon-neutral smelting conditions. They proposed adding sodium hydroxide, which binds carbon dioxide and reduces the amount of it present in the exhaust gases, to the charge. Antimony recovery remained at the level specified in other studies, but the coke content was reduced by 16–20%.

Despite the wealth of studies on this topic, including early authors’ certificates on the processing of this concentrate (which are not included in this review), researchers have only examined the distribution of the main component of sodium antimony—antimony. The behavior of other metals during reduction smelting has not been discussed.

The objective of this study was to investigate the distribution of metals present in antimony concentrate during the carbothermic reduction of sodium antimonate.

## 2. Materials and Methods

### 2.1. Materials

Sodium antimonate, an antimony-bearing concentrate (ST RK 2296-2013), was supplied by Kazzinc JSC (Ust-Kamenogorsk, Republic of Kazakhstan). According to data provided by the company, the moisture content of the concentrate was 1.95%, while our measurements indicated a value of 2.0%. The elemental composition of the initial sodium antimonate sample is presented in Table 1.

**Figure 1:**
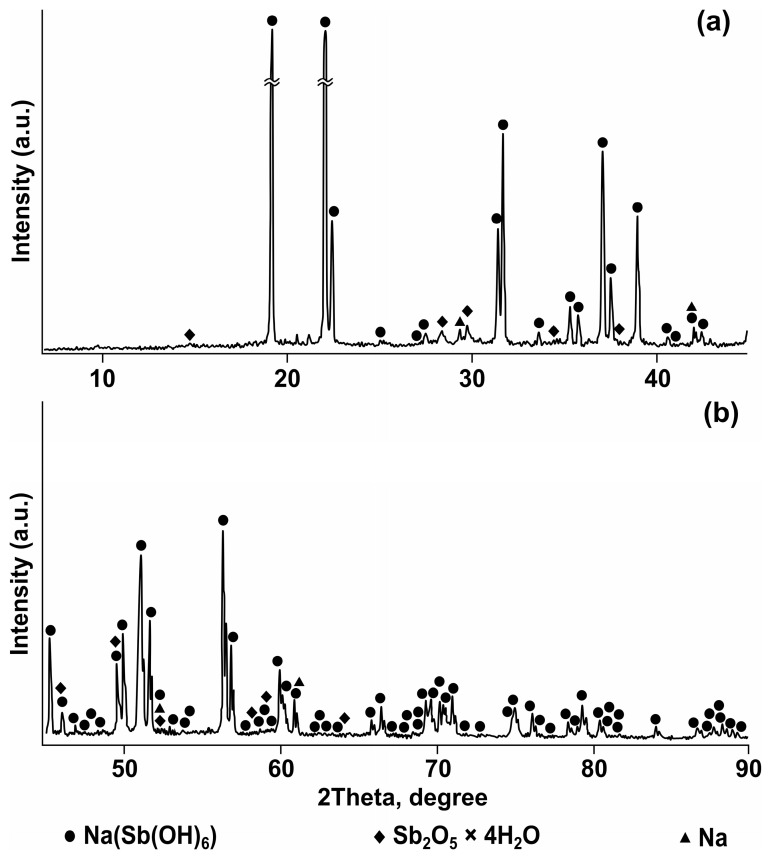
Fragments of the diffraction pattern at 2Θ = 0–45° (**a**) and 2Θ = 45–90° (**b**) for sodium antimonate (antimony concentrate).

According to X-ray phase analysis (Figure 1), the concentrate contained mopungite, or Na(Sb(OH)_6_), constituting 90.6%; pentavalent antimony oxide tetrahydrate, with the formula Sb_2_O_5_ × 4H_2_O, amounting to 5.7%; and elemental sodium, making up 3.6%.

**Figure 2:**
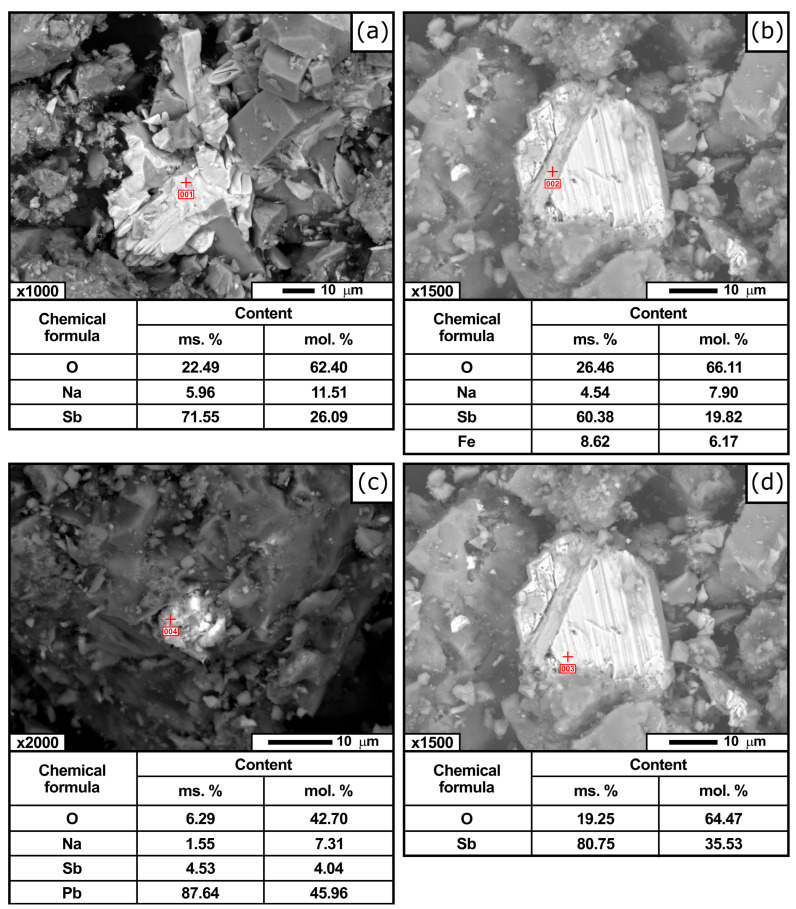
Local micrographs of the antimony concentrate: sodium antimonate and its composition (**a**); sodium antimonate with iron impurity and its composition (**b**); lead impurity in sodium antimonate and its composition (**c**); and antimony trioxide impurity in the concentrate (**d**).

Electron microscopy studies revealed that impurity elements such as sodium, lead, iron, and antimony oxides were locally distributed within the antimony concentrate (Figure 2), most likely in the form of compounds with antimony. Antimony oxides were present in hydrated form, particularly as pentavalent antimony oxide tetrahydrate (Sb_2_O_5_ × 4H_2_O).

### 2.2. Methodology

**Figure 3:**
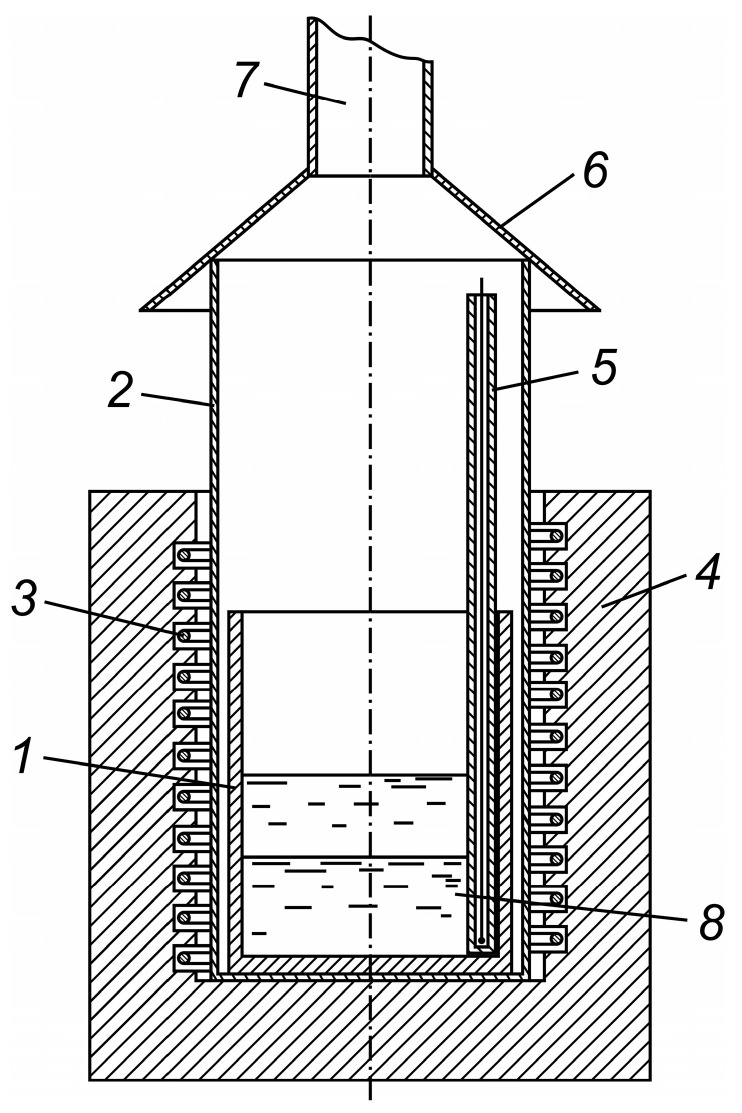
Experimental setup for smelting: (1) crucible; (2) retort; (3) electric heater; (4) shaft furnace; (5) thermocouple sheath; (6) hood; (7) exhaust pipe; and (8) layer of crude antimony.

Carbothermic reduction experiments were carried out using a shaft-type electrically heated crucible furnace with an isolated pyro-gas space. This design was necessitated by the aggressive effects of vaporized or droplet-form oxides on the heating-element material. Smelting experiments were performed using the setup schematically shown in Figure 3.

The experimental procedure was as follows. The initial materials were pre-ground to a particle size of minus 1 mm, thoroughly mixed, and charged in the required proportions into an alundum crucible (1). The crucible was then placed in a steel retort (2), which isolated the space above the crucible from the electric heater (3) located within the refractory lining grooves of the shaft furnace (4). The retort, containing the crucible, was inserted into either a cold furnace or a furnace preheated to the target experimental temperature, depending on the test conditions. A thermocouple sheath (5) was installed, with its tip positioned either inside or outside the crucible, based on prior determination of the temperature difference between the crucible and furnace space. A removable hood (6) was mounted on the upper edge of the retort to expel vapor–dust–gas emissions. The hood was freely supported on the top of the vertical retort. A pipe (7) connected the system to an exhaust hood.

The moment when the charge reached the target experimental temperature was taken to be the beginning of smelting. After a specified holding time, the hood was removed, and the thermocouple was withdrawn from the crucible. The retort, along with the crucible, was then removed from the furnace. After cooling, the smelting products were extracted from the crucible, weighed, and analyzed. Weighing was performed using JW–1 analytical balances (Acom, Pocheon, Republic of Korea) with an accuracy of ±0.1 g.

### 2.3. Characteristics

Elemental composition was determined via X-ray fluorescence (XRF) analysis using an Axios 1 kW wavelength-dispersive spectrometer (PANalytical, Almelo, The Netherlands).

Phase composition was studied via X-ray diffraction (XRD) using a D8 Advance diffractometer (Bruker, Ettlingen, Germany) with Cu-Kα radiation. Phase identification was performed using the ASTM database (reference diffraction data PDF–2 rel. 2023, ICDD, Newtown Square, PA, USA). Quantitative phase analysis was carried out using DIFFRAC.EVA software (ver. 5.2.0.5) based on the corundum number method.

Thermal analysis of the antimony concentrate sample was conducted using a simultaneous thermal analyzer (STA 449 F3 Jupiter, NETZSCH-Gerätebau GmbH, Selb, Germany). Prior to heating, the furnace chamber was evacuated and then purged with inert gas for 5 min. Heating was carried out at a rate of 15 °C/min in a high-purity argon atmosphere. The gas flow rate was maintained at 110 mL/min. The sample’s mass was 0.3 g. Data processing was performed using SCH Proteus software (ver.5.1.0/24.11.2009).

Electron microscopy studies of sodium antimonate before and after smelting were carried out using a JSM–8230 electron probe microanalyzer (JEOL, Tokyo, Japan).

## 3. Results and Discussion

### 3.1. Thermal Analysis of the Initial Antimony Concentrate

**Figure 4:**
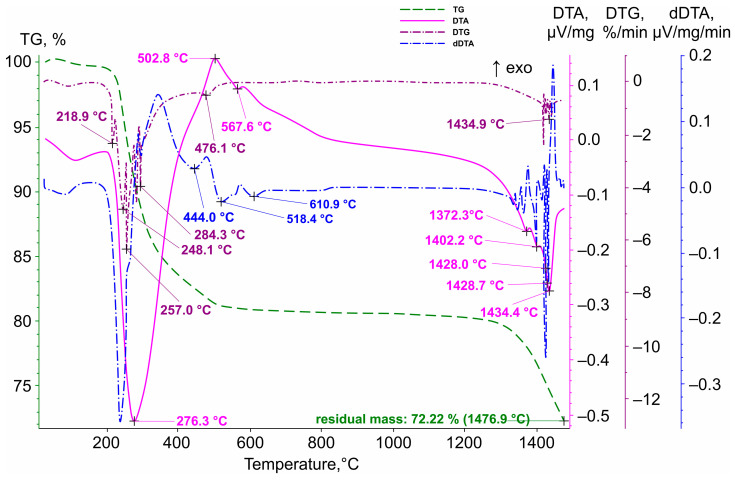
Thermogravimetric analysis of the antimony concentrate.

The results of the thermal analysis of sodium antimonate are presented in Figure 4. As shown, the DTA curve exhibits endothermic effects with maxima at 273.6 °C, 567.6 °C, 1372.3 °C, 1402.2 °C, 1426 °C, 1428.7 °C, and 1434.4 °C. An exothermic effect can also be observed at 502.8 °C. The dDTA curve reveals additional endothermic effects with extrema at 440 °C, 518.4 °C, and 610.9 °C.

The combination of a pronounced endothermic effect with a maximum at 273.6 °C, accompanied by a decrease in sample mass, and an exothermic effect with a peak at 585.1 °C can tentatively be attributed to the behavior of NaSb(OH)_6_. In the temperature range around 273.6 °C, dehydration occurs, while the exothermic peak likely reflects the crystallization of a new phase. The endothermic effect with an extremum at 440 °C is also accompanied by a decrease in sample mass; on the DTG curve, it corresponds to a weak minimum at 476.1 °C. This may indicate dehydration of iron hydroxide impurities. In the region of the weak endothermic effect with a maximum at 567.6 °C, several processes may have occurred, including a peritectic transformation in the Sb–Te system. According to different sources, this transformation occurs at either 557.2 °C or 548 °C. Additionally, transformations may occur in the Fe–Sb–S and Pb–Sb–S systems, namely, FeSb_2_S_4_ → FeS + melt and Pb_6_Sb_10_S_21_ + Pb_5_Sb_4_S_11_ → Pb_5_Sb_6_S_14_, respectively.

The weak endothermic effect with an extremum at 518.4 °C, on the dDTA curve, may correspond to a transformation in the Fe–Sb–S system: Sb_2_S_3_ + Sb + FeSb_2_S_4_ → melt. The weak endothermic effect at 610.9 °C on the dDTA curve may be associated with melting of Sb_2_Te_3_ and/or eutectic melting in the As–Sb system.

A series of endothermic effects in the temperature range of 1300–1477 °C most likely corresponds to the stepwise melting of the sodium antimonate sample. Simultaneously, volatilization of Sb_2_O_3_ occurs.

### 3.2. Reduction Smelting of Antimony Concentrate

To determine the optimal charge composition, preliminary studies were conducted to evaluate the effect of reductant consumption on the yield of crude antimony in the metallic phase. Coke breeze (corresponding to a fraction of <1 mm of crushed coke) with an ash content of 15.33% was used as the reducing agent. Experiments were carried out at a temperature of 900 °C with coke additions of 5, 10, and 15 wt.% relative to the charged sodium antimonate. The holding time after the target temperature was reached was 1 h. The initial sample mass ranged from 70.0 to 500 g. The results of the experiments are presented in Table 2.

**Figure 5:**
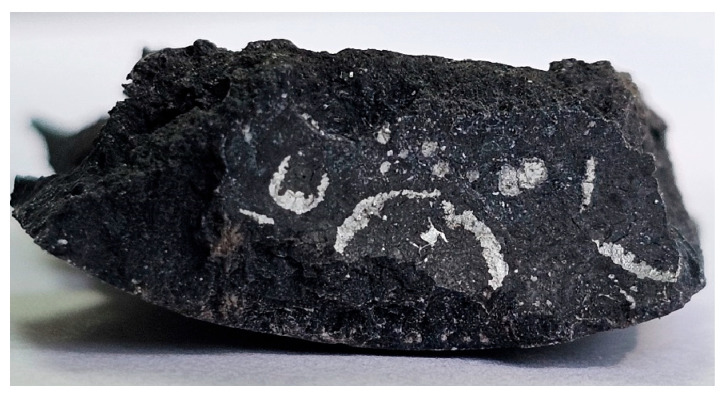
Antimony inclusions in slag.

It can be observed that an increase in the amount of reductant is accompanied by an increase in the yield of the metallic phase (crude antimony). The antimony content in the crude metal passes through a minimum (87.32%) at 10 wt.% of reductant, while Sb recovery reaches a maximum (71.49%) at the same concentration. With increasing coke consumption, the amount of slag and the antimony content in it decrease from 55.83% to 0.56%. At the same time, the sodium content in the crude antimony increases significantly, reaching up to 5.67%. This effect is likely associated with the highly reducing atmosphere of the process. At low reductant consumption, a significant amount of antimony remains in the slag, and this amount is visible (Figure 5). Thus, the optimal reductant consumption is 10 wt.%.

To study the distribution of metals among the products of carbothermic reduction, smelting experiments were conducted at temperatures of 800, 900, and 1000 °C. The reductant consumption was fixed at 10% of the sodium antimonate mass. The holding time was 1 h after reaching the target temperature. During analysis of the experimental data, the distribution of antimony, arsenic, and sodium was evaluated.

At 800 °C, no complete melting of the charge was observed; however, droplets of metallic antimony were present on the surface. The results of sodium antimonate smelting with coke at temperatures of 900 and 1000 °C are presented in Table 3 and Table 4.

Analysis of the results indicates that the amount of antimony recovered as crude metal during reduction smelting is comparable to industrial production levels [14]. At 900 °C, 72.35% of antimony is recovered as crude metal, with an Sb content of 94.05%. When the temperature is increased to 1000 °C, the indicators change only slightly: the antimony content in the crude metal decreases to 87.32%, with recovery at 71.49%.

A significant amount of sodium was found both in the initial concentrate and in the smelting products. The majority of sodium (more than 80%) was transferred to the slag phase. Its content in crude antimony after smelting was in the range of 0.68–0.69%. Arsenic is distributed between both smelting products. At the same time, a lower arsenic content was observed in the slag obtained at 1000 °C, which is likely due to its partial volatilization.

**Figure 6:**
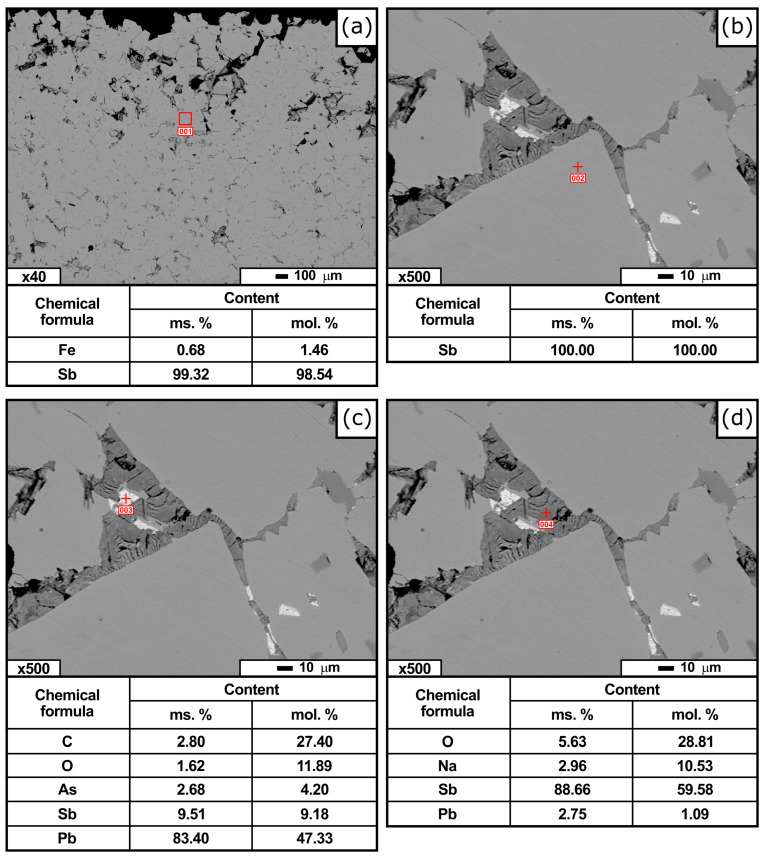
Micrographs of the metallic phase and elemental distribution (SEM analysis): metallic antimony with iron impurity (**a**); crude antimony matrix (**b**); impurity inclusion with predominance of lead in the antimony matrix (**c**); impurity inclusion containing lead and sodium (**d**).

**Figure 7:**
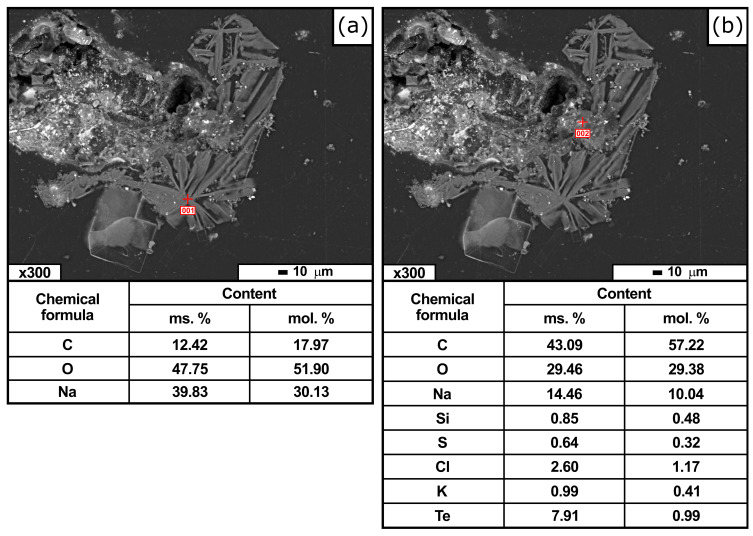
Micrographs of the slag phase and elemental distribution (SEM analysis): slag phase (**a**); slag phase with impurities (**b**).

Electron microscopy studies of the products of carbothermic reduction, including their composition and microanalysis locations, are presented in Figure 6 and Figure 7.

The structure of the metallic phase was predominantly homogeneous (Figure 6) and consisted of grains with well-defined boundaries. The grain size ranged from approximately 20 to 80 μm. Analysis of local regions of the matrix (Figure 6a) showed that it consisted primarily of antimony with iron impurities, the content of which varied from 0.05 to 0.99 wt.% depending on the sampling location. Point microanalysis (Figure 6b) revealed that the matrix was composed of nearly pure antimony, while iron occurred as compositionally heterogeneous inclusions within the matrix, which also contained antimony and arsenic.

Heterogeneous inclusions were also identified at grain boundaries. Figure 6c shows the inclusions, predominantly lead, while carbon, antimony, and arsenic are also present. Microprobe analysis of a neighboring inclusion (Figure 6d) revealed the following composition: 88.66 wt.% Sb, 2.96 wt.% Na, and 2.75 wt.% Pb. In both cases, a significant amount of oxygen (2.8–5.63 wt.%) was detected in the impurity inclusions.

Similar studies of the slag from reduction smelting showed that it consisted either of a single phase of sodium carbonate (Figure 7a) or sodium carbonate with various impurities (Figure 7b). Notable features include the presence of tellurium in the slag at a concentration of 7.91 wt.% and its absence in crude antimony, indicating preferential transfer of Te to the slag phase.

Thus, during reduction smelting of sodium antimonate, antimony forms a metallic phase with an Sb content of 87.32–94.5 wt.% and a recovery of 71.49–72.35%. These values correspond to recovery levels typical of industrial processes.

A significant amount of sodium was found in both the initial concentrate and the smelting products, with more than 80% found in the slag. The sodium content in crude antimony after smelting was 0.68–0.69%. Lead was predominantly concentrated in crude antimony, while iron was also present in the metallic phase, as expected. Arsenic was distributed between the metallic and slag phases. Other impurities were locally distributed in both the metallic phase and the slag.

## 4. Conclusions

As a result of the carbothermic reduction of sodium antimonate—an intermediate product of lead refining—in crucible smelting, it was established that the optimal process temperature is 900 °C with 10% coke consumption (and an ash content up to 15.33%). Under these conditions, the amount of antimony recovered as crude metal reaches 71–72%, a range comparable to industrial performance levels [14]. The process does not involve the addition of slag-forming components. The antimony content in the crude metal reaches up to 94.5%. A significant portion of antimony (up to 27%) volatilizes with off-gases.

Sodium participates in the formation of the slag phase, with more than 80% becoming slag. A considerable amount of sodium is also present in crude antimony, reaching up to 8% in individual samples.

Arsenic is distributed among the metallic, slag, and gas phases.

Lead is predominantly accumulated in the liquid antimony phase. Other impurities are distributed in comparable amounts between the metallic and slag phases. Iron preferentially dissolves in crude antimony.

Electron microscopy studies revealed that impurities are locally distributed in both crude antimony and slag, often forming heterogeneous clusters composed of elements with varying compositions and concentrations.

Tellurium present in sodium antimonate was detected in certain slag samples.

These results improve our understanding of the carbothermic reduction of sodium antimonate.

## Figures and Tables

**Table 1:** Elemental composition of the antimony concentrate according to data from IMOB JSC.

Elements	Sb	As	Sn	Pb	Na	Te	Fe	Al	S	Ca	Zn	Mo	In	O
Content, wt.%	60.39	0.60	-	0.56	7.66	0.79	0.52	0.01	0.04	0.18	0.02	0.13	0.17	28.93

**Table 2:** Effect of reductant amount on smelting product yield, crude antimony yield, and antimony recovery.

Parameter	Coke Consumption Relative to Sodium Antimonate, wt.%		
	5	10	15
Crude antimony yield relative to the charged feed, wt.%	19.57	38.60	40.14
Antimony content in crude antimony, wt.%	97.90	87.32	92.42
Slag yield relative to the charged feed, wt.%	49.39	21.01	3.73
Antimony content in slag, wt.%	55.83	29.55	0.56
Sodium content in rough antimony, wt.%	0.25	0.69	5.67
Extraction of antimony into blister metal, wt.%	30.67	71.49	59.56

**Table 3:** Distribution of elements during smelting of sodium antimonate at 900 °C.

Balance Sheet Items	Quantity		Sb		Na		As	
	g	%	Content, %	Recovery, %	Content, %	Recovery, %	Content, %	Recovery, %
Loaded								
Sb concentrate	73.4	90.84	60.39	100	7.66	100	0.60	100
Breeze coke	7.4	9.16	–	–	–	–	–	–
Total:	88.8	100	–	100	–	100	–	100
Received								
Sb rough	34.1	42.20	94.05	72.35	0.68	4.12	0.50	38.71
Slag	19.7	24.38	1.32	0.59	30.84	108.07	0.51	33.81
Total	53.8	66.58	–	72.94	–	112.19	–	61.52
Losses with gases and balance residuals	27.0	−33.42	–	−27.06	–	+12.19	–	−38.48

**Table 4:** Distribution of elements during smelting of sodium antimonate at 1000 °C.

Balance Sheet Items	Quantity		Sb		Na		As	
	g	%	Content, %	Recovery, %	Content, %	Recovery, %	Content, %	Recovery, %
Loaded								
Sb concentrate	81.2	90.75	58.06	100	8.13	100	0.65	100
Breeze coke	8.3	9.27	–	–	–	–	–	–
Total:	89.5	100	–	100	–	100	–	100
Received								
Sb rough	38.6	43.13	87.32	71.49	0.69	4.03	0.48	35.39
Slag	18.8	21.01	0.99	3.95	29.55	84.15	0.43	15.31
Total	57.4	64.14	–	75.44	–	88.12	–	50.70
Losses with gases and balance residuals	−32.1	–35.86	–	−24.56	–	−11.82	–	−49.30

## Data Availability

The original contributions presented in this study are included in the article. Further inquiries can be directed to the corresponding author.
